# Prognostic significance of the pretreatment serum gamma-glutamyltransferase levels in Chinese patients with non-metastatic cervical cancer

**DOI:** 10.18632/oncotarget.22273

**Published:** 2017-11-01

**Authors:** Yi Zhu, Ai-Jun Zhang, Da-Bao Wu, Zhen Shen, Gang Chen, Yang-Yang Shi, Hao Wu, Jing Wang

**Affiliations:** ^1^ Department of Obstetrics and Gynecology, Anhui Provincial Hospital Affiliated Anhui Medical University, Hefei, Anhui, 230001, People’s Republic of China

**Keywords:** cervical cancer, serum gamma-glutamyltransferase, GGT, survival

## Abstract

This study was performed to evaluate the prognostic significance of the pretreatment serum gamma-glutamyltransferase (GGT) levels in a Chinese cohort of patients with early-stage or locally advanced cervical cancer. The pretreatment serum GGT levels were examined in 290 cervical cancer patients with stage I-III disease and 230 healthy controls selected from a cancer-free population in the same region. Patients were assigned to normal or high-risk GGT groups, as previously described, and the GGT levels were correlated to clinicopathologic parameters and survival data. The GGT levels in cervical cancer patients were significantly higher than those in healthy controls (35.6 ± 29.1 vs. 24.1 ± 14.7 U/L, *P* < 0.001). In addition, the pretreatment serum GGT levels were associated with the histology type (*P* = 0.023), lymph node involvement (*P* = 0.040), stage (*P* = 0.029), recurrence (*P* = 0.015) and death (*P* = 0.005), but not with age (*P* = 0.432), tumor size (*P* = 0.067) or degree of differentiation (*P* = 0.901). Moreover, univariate survival analysis revealed that patients with high GGT levels tended to have poorer disease-free survival (DFS) [hazard ratio (HR), 1.721; 95% confidence interval (CI), 1.189–2.491; *P* = 0.004] and overall survival (OS) (HR, 1.929; 95% CI, 1.294–2.876; *P* = 0.001) compared to those with normal GGT levels. However, a multivariate Cox-regression model did not support these data (HR, 1.373; 95% CI, 0.925–2.039; *P* = 0.116 for DFS and HR, 1.357; 95% CI, 0.887–2.078; *P* = 0.160 for OS, respectively) after adjusting for other confounding variables. High pretreatment serum GGT was associated with more advanced tumor behavior, but could not serve as an independent prognostic indicator in patients with early-stage or locally advanced cervical cancer.

## INTRODUCTION

Cervical cancer is one of the most commonly diagnosed cancers and is high in incidence and mortality among women worldwide. The morbidity and mortality in developed countries have decreased dramatically as a result of thorough screening with Papanicolaou tests, early treatment of preinvasive lesions, as well as vaccination against human papillomavirus (HPV) [1–5]. However, in many developing regions, cervical cancer remains a major cause of death in women, with approximately 530,000 new cases and 275,000 deaths annually [2]. Although early-stage and locally advanced cervical cancer can be cured with radical surgery, chemoradiotherapy, or a combination of these treatments, patients with metastatic or recurrent disease following platinum-based chemoradiotherapy have limited options [6–7], and the prognosis remains poor. Several markers have recently been proposed as potential prognostic factors, including squamous cell carcinoma antigen, cancer antigen-125 and plasma fibrinogen [8]. However, there is still a lack of an optimal indicator to estimate the recurrence risk and outcome in patients with early-stage or locally advanced cervical cancer.

Gamma-glutamyltransferase (GGT) is a membrane-bound enzyme that is involved in glutathione (GSH) metabolism by transferring gamma-glutamyl functional groups. GSH has been identified as a major water-soluble antioxidant in cells and protects cells against oxidants by neutralizing reactive oxygen compounds and free radicals that are produced during normal metabolism [9–10]. Thus, an increase in GGT and GSH levels is frequently observed in pathological states of oxidative stress [10–11]. Moreover, in addition to serving as a routine marker for hepatobiliary disease [9, 12], GGT can also modulate the cellular proliferative and apoptotic balance and plays an important role in cancer development, progression, invasion, and anticancer drug resistance [13–16]. Recently, GGT has gained increased attention as an independent prognostic biomarker in various malignancies, including renal cell carcinoma, ovarian cancer, endometrial cancer, as well as esophageal squamous cell carcinoma [17–19]. Very recently, high pre-therapeutic GGT levels have been identified to be associated with advanced tumor stages, but did not predict survival in patients with cervical cancer [20].

Therefore, the aim of the present study was to investigate the potential prognostic significance of the pretreatment serum GGT levels in a large Chinese cohort of patients with early-stage or locally advanced cervical cancer.

## RESULTS

### Serum GGT levels in cervical cancer patients and healthy controls

**Figure 1:**
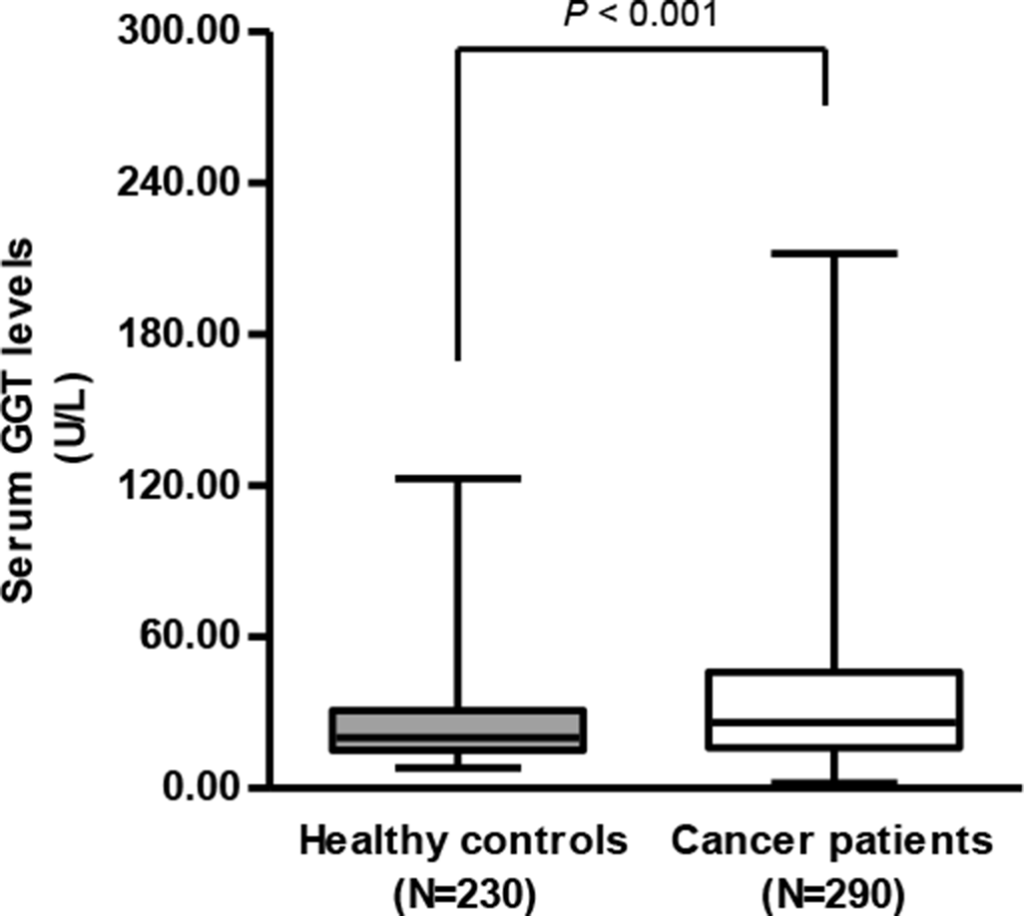
Serum GGT levels in cervical cancer patients (*N* = 290) was significantly higher than those of healthy controls (*N* = 230) (35.6 ± 29.1 vs. 24.1 ± 14.7 U/L, student’s *t*-test, *P* < 0.001)

A consecutive cohort of 290 patient with stage I-III cervical cancer and 230 healthy controls were enrolled in the study. The median age was 45 (range, 20–81 years) for cancer patients and 46 (range, 21–80) for control subjects. No statistically significant differences in age, gender and comorbidities were found between the patients and the healthy subjects. The serum GGT levels were significantly higher in cervical cancer patients than those of healthy controls (35.6 ± 29.1 vs. 24.1 ± 14.7 U/L, *P* < 0.001, Figure 1). Moreover, high-risk GGT levels were more frequently seen in cervical cancer patients compared to the control subjects (36.2% vs 14.3%).

### Patient demographics

Of the 290 included patients, 272 (93.8%) patients had squamous cell carcinoma, while 18 (6.2%) had non-squamous cell carcinoma. A total of 145 (50.0%) patients were classified as stage I, 101 (34.8%) as stage II and 44 (15.2%) as stage III. The number of patients with poorly, moderately and well-differentiated tumors was 65 (22.4%), 181 (62.4%) and 44 (15.2%), respectively. The clinicopathologic parameters of the 290 patients are presented in Table 1.

**Table 1:** The association of the pretreatment serum GGT levels with clinicopathologic characteristics in 290 non-metastatic cervical cancer patients

Clinicopathologiccharacteristics	Patients*N* (%)	Serum GGT level (*N*, %)		*P* value
		Normal (< 36.0 U/L)	High (≥ 36.0 U/L)	
Age (years)				0.432
< 45	122 (42.1)	81 (43.8)	41 (39.0)	
≥ 45	168 (57.9)	104 (56.2)	64 (61.0)	
Tumor size (cm)				0.067
< 4	145 (50.0)	100 (54.1)	45 (42.9)	
≥ 4	145 (50.0)	85 (45.9)	60 (57.1)	
Histology type				0.023^*^
SCC	272 (93.8)	178 (96.2)	94 (89.5)	
Non-SCC	18 (6.2)	7 (3.8)	11 (10.5)	
Degree of Differentiation				0.901
G1	44 (15.2)	28 (15.1)	16 (15.2)	
G2	181 (62.4)	114 (61.6)	67 (63.8)	
G3	65 (22.4)	43 (23.2)	22 (21.0)	
Lymph node involvement				0.040^*^
Negative	180 (62.1)	123 (66.5)	57 (54.3)	
Positive	110 (37.9)	62 (33.5)	48 (45.7)	
FIGO Stage				0.029^*^
I	145 (50.0)	103 (55.7)	42 (40.0)	
II	101 (34.8)	59 (31.9)	42 (40.0)	
III	44 (15.2)	23 (12.4)	21 (20.0)	
Recurrence				0.015^*^
No	176 (60.7)	122 (65.9)	54 (51.4)	
Yes	114 (39.3)	63 (34.1)	51 (48.6)	
Death				0.005^*^
No	193 (66.6)	134 (72.4)	59 (56.2)	
Yes	97 (33.4)	51 (27.6)	46 (43.8)	

GGT, gamma-glutamyltransferase; SCC, squamous cell carcinoma; FIGO, international federation of gynecology and obstetrics.

^*^ *P* < 0.05.

### Association between serum GGT levels and clinicopathologic parameters

The relationship between serum GGT levels and clinicopathologic features is summarized in Table 1. The results indicated that a high-risk GGT group affiliation was significantly associated with the histology type (*P* = 0.023), lymph node involvement (*P* = 0.040), FIGO stage (*P* = 0.029), recurrence (*P* = 0.015) and death (*P* = 0.005) but not with patients' age (*P* = 0.432), tumor size (*P* = 0.067) or degree of differentiation (*P* = 0.901).

### Prognostic significance of the pretreatment serum GGT levels

**Figure 2:**
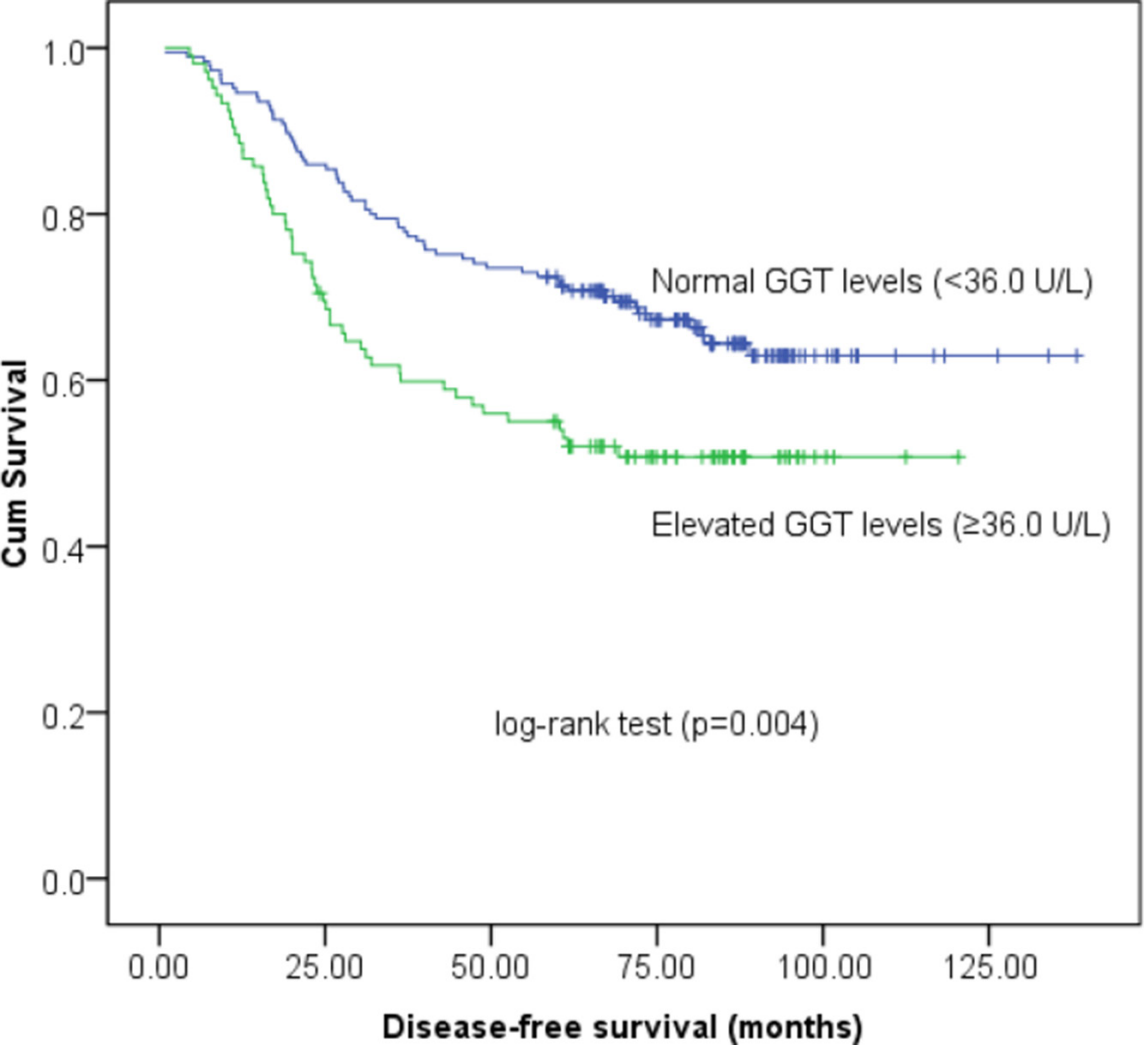
Kaplan-Meier survival estimates of disease-free survival (DFS) for 290 cervical cancer patients according to GGT groups

**Figure 3:**
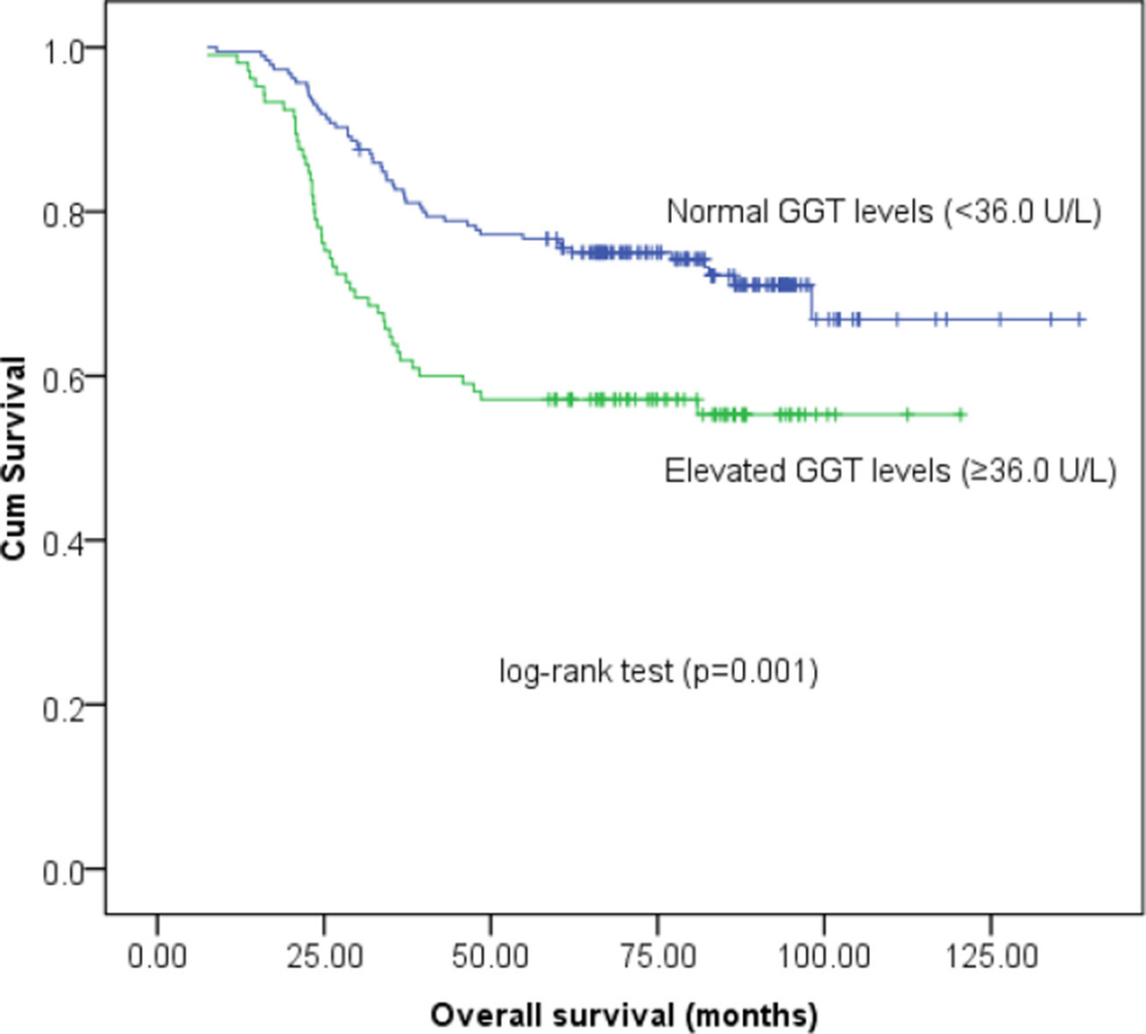
Kaplan-Meier survival estimates of overall survival (OS) for 290 cervical cancer patients according to GGT groups

Univariate survival analysis of DFS demonstrated that the high-risk GGT group was significantly more likely to experience reduced DFS (HR, 1.721; 95% CI, 1.189–2.491; *P* = 0.004; Figure 2) than those with normal serum GGT levels. Tumor size (< 4/≥ 4 cm), lymph node involvement (Negative/Positive) and tumor stage (I/II/III) also constituted significant prognostic variables, as identified by univariate analysis (*P* < 0.05, Table 2). Moreover, Kaplan-Meier survival analysis of OS indicated that patients with high-risk GGT levels tended to have lower OS rates (HR, 1.929; 95% CI, 1.294–2.876; *P* = 0.001; Figure 3). In addition, other parameters, including tumor size, lymph node involvement and tumor stage was also significantly predictive of OS in cervical cancer patients (Table 3). However, the multivariate Cox-regression model analysis of DFS and OS failed to identify baseline serum GGT levels as an independent prognostic indicator (HR, 1.373; 95% CI, 0.925–2.039; *P* = 0.116 for DFS and HR, 1.357; 95% CI, 0.887–2.078; *P* = 0.160 for OS, respectively, Tables 2 and 3) after adjusting for other confounding variables. As expected, tumor size, lymph node involvement and tumor stage were identified as significant prognostic variables for both DFS and OS.

**Table 2:** Univariate and multivariate analysis of DFS in 290 cervical cancer patients

Variables	Univariate			Multivariate		
	HR	95% CI	*P*	HR	95% CI	*P*
Age (< 45 vs. ≥ 45 years)	1.286	0.874–2.156	0.283	1.246	0.818–1.194	0.267
Tumor size (≥ 4.0 vs. < 4.0 cm)	3.687	2.442–5.566	< 0.001^*^	2.444	1.599–3.737	< 0.001^*^
Histology type (SCC vs. Non-SCC)	1.474	0.746–2.911	0.264	1.401	0.683–2.876	0.358
Degree of differentiation (G1 vs. G2 vs. G3)	0.862	0.640–1.160	0.326	0.841	0.596–1.186	0.323
Lymph node involvement (Positive vs. Negative)	2.418	1.672–3.496	< 0.001^*^	1.579	1.080–2.308	0.019^*^
Tumor stage (FIGO III vs. II vs. I)	4.116	3.686–6.982	< 0.001^*^	4.029	2.492–6.093	< 0.001^*^
GGT groups (High vs. Normal)	1.721	1.189–2.491	0.004^*^	1.373	0.925–2.039	0.116

DFS, disease-free survival; HR, hazard ratio; CI, confidence interval.

^*^ *P* < 0.05.

**Table 3:** Univariate and multivariate analysis of OS in 290 cervical cancer patients

Variables	Univariate			Multivariate		
	HR	95% CI	*P*	HR	95% CI	*P*
Age (< 45 vs. ≥ 45 years)	1.387	0.796–1.983	0.346	1.046	0.683–1.342	0.423
Tumor size (≥ 4.0 vs. < 4.0 cm)	4.356	2.766–5.584	< 0.001^*^	3.298	1.986–4.877	< 0.001^*^
Histology type (SCC vs. Non-SCC)	1.701	0.857–3.379	0.264	1.321	0.637–2.740	0.455
Degree of differentiation (G1 vs. G2 vs. G3)	0.935	0.674–1.299	0.690	1.046	0.724–1.513	0.810
Lymph node involvement (Positive vs. Negative)	2.707	1.810–4.049	< 0.001^*^	1.591	1.045–2.423	0.030^*^
Tumor stage (FIGO III vs. II vs. I)	4.491	3.129–6.316	< 0.001^*^	3.618	2.531–4.912	< 0.001^*^
GGT groups (High vs. Normal)	1.929	1.294–2.876	0.001^*^	1.357	0.887–2.078	0.160

OS, overall survival.

^*^ *P* < 0.05.

## DISCUSSION

A possible relationship between serum GGT and the incidence, as well as the prognosis, of various malignancies such as hepatocellular carcinoma, esophageal squamous cell carcinoma and many gynecological tumors has gained increased attention [17–20]. Recently, Strasak et al. reported a statistically significant association between GGT and invasive cervical cancer risk. In addition, they identified GGT as a prognostic marker for cervical cancer, suggesting that increased levels of serum GGT were associated with an increased risk of the progression of premalignant cervical lesions to invasive cancer [21]. However, published data on the potential prognostic impact of serum GGT are very limited.

In a recent retrospective analysis, Polterauer et al. assigned 692 patients with cervical cancer to previously described GGT risk groups and performed uni- and multivariate survival analysis. They determined that the GGT serum levels were associated with FIGO stage and age but not with lymph node involvement or histology type. Moreover, a high-risk GGT group affiliation was significantly associated with poor DFS and OS in a univariate analysis. However, this finding was not confirmed in a multivariate survival analysis, likely due to the strong association between tumor stage and GGT [20]. Therefore, they suggested that GGT may serve as a marker of disease progression, rather than as an independent prognostic indicator. Nevertheless, in accordance with their findings, the results from the present analysis of 290 patients with early-stage or locally advanced cervical cancer revealed that subjects with an elevated (≥ 36.0 U/L) serum GGT level prior to treatment had substantially shorter DFS and OS as indicated by the univariate analysis. Unfortunately, we failed to demonstrate the baseline serum GTT level as an independent prognostic variable in this group of patients. Moreover, the preoperative serum GGT levels did not represent an independent prognostic factor in a European cohort of patients with non-metastatic renal cell carcinoma [22].

Serum GGT is routinely used as a sensitive indicator of hepatobiliary disorder and a marker of alcohol intake in clinical practice [9, 12]. In addition, its ability to regulate redox-sensitive functions, including cellular proliferation and apoptotic balance, as well as antioxidant effects has been confirmed. Moreover, it has also been suggested to play a role in tumor progression, invasion, and drug resistance [13–16]. However, the specific mechanisms by which GGT becomes elevated in cancer patients remains poorly understood. As previously described, GSH is crucial in the removal and detoxification of various carcinogens by conjugating with them in the extracellular microenvironment [9–11, 23]. As cellular GGT is indispensable for the metabolism of extracellular GSH conjugates, higher serum GGT levels reflect more xenobiotics that require conjugation and the corresponding increased cellular GGT activity to metabolize them [21]. Thus, the elevation of GGT may be an indicator in the development of an aggressive disease [20]. Moreover, researchers have demonstrated that serum GGT elevation may act as a part of the activation of the host immune response as a mechanism of immune-mediated cancer rejection. Meanwhile, the tumor itself may also produce GGT, leading to increased circulating GGT levels in the serum [11, 16]. Further research is warranted in order to elucidate the exact biological mechanisms linking serum GGT levels to advanced tumor behavior.

As with all retrospective studies, the main limitations of this study were the retrospective design, the long study period and the multiple surgeons involved. Despite these limitations, our study demonstrated that high serum GGT levels prior to treatment were associated with advanced tumor behavior but could not serve as an independent prognostic factor in patients with early-stage or locally advanced cervical cancer. Large-scale prospective studies are needed before baseline serum GGT levels can be introduced as a routine marker in specific patients.

## MATERIALS AND METHODS

### Patients

**Figure 4:**
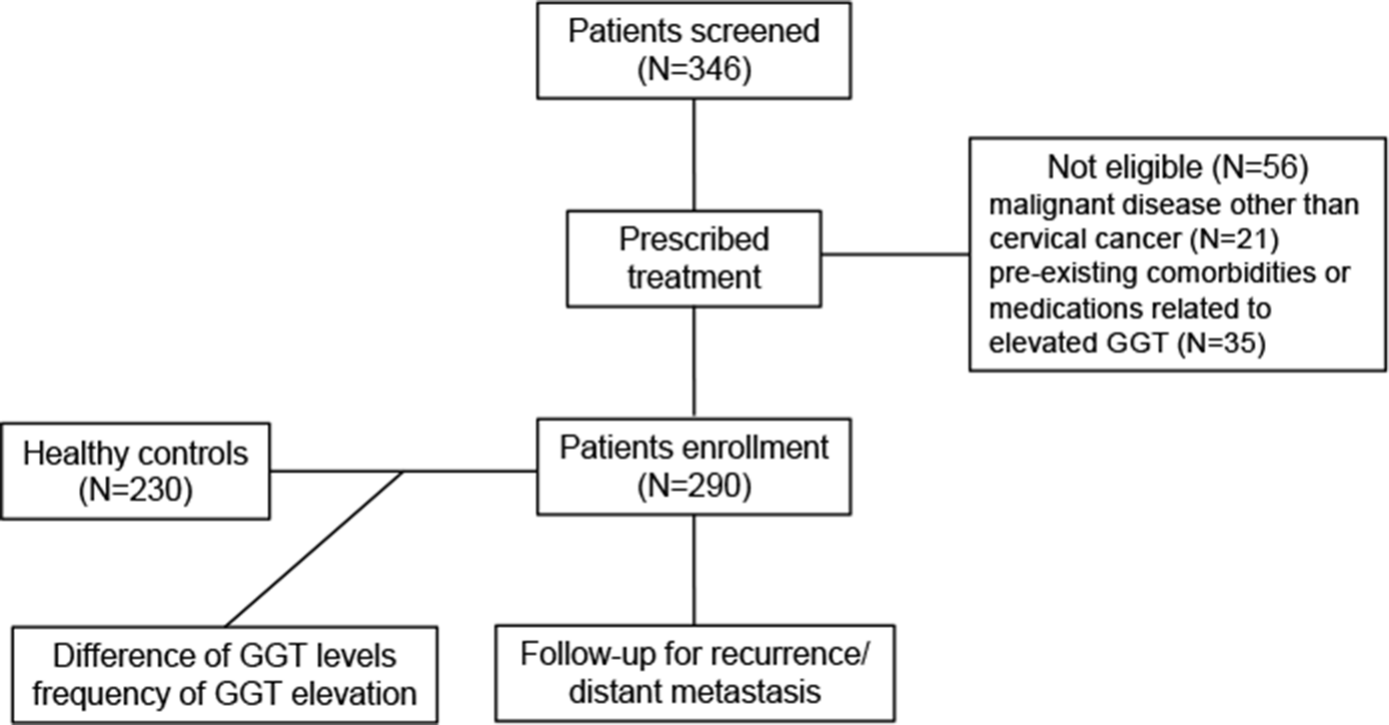
The flowchart of this study GGT, gamma-glutamyltransferase.

A total of 290 patients with early-stage or locally advanced cervical cancer who were treated in the Department of Obstetrics and Gynecology at the Anhui Provincial Hospital Affiliated Anhui Medical University (Hefei, China) between January 2005 and December 2010 were included in this study. Patients with malignant disease other than cervical cancer and those presenting with pre-existing comorbidities or medications related to elevated GGT (i.e., hepatobiliary tract, pancreatic, and heart disease or alcohol abuse) were excluded from the study. Healthy controls were selected from a cancer- and hepatobiliary disease-free population in the same region for comparison of GGT levels (Figure 4).

Briefly, subjects with microinvasive disease were treated with conization or hysterectomy combined with systematic pelvic/periaortic lymphadenectomy. Radical hysterectomy or trachelectomy with systematic pelvic/periaortic lymphadenectomy was utilized to treat those with early-stage disease. Locally advanced cases were concurrently treated with radiochemotherapy. Follow-up occurred for all patients every 3 months for the first 2 years after the initial treatment, every 6 months in the third year and yearly thereafter. A physical examination, ultrasonography and computed tomography were performed and tumor markers were evaluated during the follow-up period. Written informed consent was obtained. The study was approved by the independent ethics committee at the Anhui Provincial Hospital and was performed in accordance with the Declaration of Helsinki.

### Clinical and laboratory parameters

Patients' baseline characteristics, including the clinical evaluations, laboratory test results, pathological conditions, treatments, and follow-up data were retrospectively reviewed and extracted from patient registries. All patients were managed according to the international guidelines. The International Federation of Gynecology and Obstetrics criteria (FIGO 2009) staging system was utilized to classify the tumor stage. The tumor size was defined according to the longest diameter measured using the post-operative pathological specimens. The degree of differentiation was categorized as poorly, moderately or well-differentiated. Serum GGT levels were determined with an enzyme kinetic assay using the optimal cutoff value of 36.0 U/L, as previously described. In this manner, patients were divided into two risk groups: a high-risk GGT group (≥ 36.0 U/L) and a normal GGT group (< 36.0 U/L). Disease-free survival (DFS) was calculated from the date of surgery to local recurrence/distant metastasis or to the last date of follow-up. Overall survival (OS) was defined as the time interval from the date of surgery to death from cervical cancer or to the last date of follow-up.

### Statistical analysis

Age and GGT levels in the patient and control samples were compared using Student’s *t*-test. Serum GGT concentration was analyzed as a categorical variable, after grouping with the threshold of 36.0 U/L. The chi-square test or Fisher’s exact test was applied to determine the different distribution of baseline and clinicopathologic parameters between groups. The Kaplan-Meier method was utilized to calculate survival curves, and survival differences were compared with the log-rank test. Cox proportional hazards models were used for univariate and multivariate analysis to determine hazard ratios (HRs) for the DFS- and OS-related variables. HRs with 95% confidence intervals (CIs) and two-sided *P* values were reported. All statistical analyses were performed with SPSS 23.0 (SPSS Inc., Chicago, IL, USA). A two-sided *P* value of less than 0.05 was considered statistically significant.
